# Donor human milk: actuality and perspectives

**DOI:** 10.1186/1824-7288-41-S2-A7

**Published:** 2015-09-30

**Authors:** Enrico Bertino, Chiara Peila, Alessandra Coscia, Melissa Raia, Ilaria Rovelli, Claudia Rossi, Natascia Cinatti

**Affiliations:** 1 Neonatology Unit, Department of Public Health and Pediatrics, University of Turin, Italy

## 

Growing clinical evidences indicate the benefits of human milk (HM) for appropriate growth and development of a newborns: the particular composition make it a unique and inimitable nutrient [1]. Mother's own milk is the first choice for all neonates including preterm infants, when it is unavailable or in short supply, donor milk (DM) is an important alternative. DM should be provided from an established Human Milk Bank (HMB), which follows specific safety guidelines [2]. When HMB is available, significantly less neonatal intensive care unit (NICU) neonates receive formula milk in the first weeks of life. In Italy, data from NICU patients show that exclusive breastfeeding at discharge is achieved for nearly 30% of neonates when banked milk is available during hospitalization and only for 16% of neonates when it is not [3]. DM banking should be promoted, protected, and supported as an extension of national breastfeeding policies, in this regard Italian National Guidelines were published on “Gazzetta Ufficiale della Repubblica Italiana” in 2014 [4].

Nonetheless storage and processing of human milk may reduce some biological components, which may diminish its health benefits but when donor milk is used instead of formula, it is demonstrated a reduction in the incidence of necrotizing enterocolitis and an enhanced feeding tolerance [5]. Pasteurization of the milk is necessary for minimize the risk of disease transmission, inactivating most of the viral and bacterial contaminants. Holder pasteurization (62.5°C for 30 minutes) is the most commonly used method and actually allows a good compromise between microbiological safety and nutritional and biological quality of the milk [2,4]. It results in the loss of the quantity and/or activity of some biologically functional milk components to varying degrees. The optimal pasteurization process should be optimized to maintain microbiological safety while preserving the highest amount and activity of the bioactive milk components. New methods to improve the biological quality and safety of DM are under investigation. High-temperature short-term pasteurization (flash pasteurization, 72°C for 5-15 seconds) and its homemade low-tech variant for developing countries (flash-heat treatment), thermoultrasonic treatment, high-pressure processing are the alternative methods on which present studies are focused.

Future research should focus on the improvement of milk processing in HMB, particularly of heat treatment on the optimization of DM fortification and on further evaluation of the potential clinical benefits of processed and fortified DM.
